# Crystal structure of 2-chloro-1-(3-methyl-2,6-di­phenyl­piperidin-1-yl)ethanone

**DOI:** 10.1107/S205698901500122X

**Published:** 2015-01-28

**Authors:** V. Shreevidhyaa Suressh, K. Prathebha, S. Abdul Basheer, S. Ponnuswamy, G. Usha

**Affiliations:** a Department of Physics, Anna Adarsh College for Women, Chennai-40, Tamilnadu, India; b PG and Research Department of Physics, Queen Mary’s College, Chennai-4, Tamilnadu, India; c PG and Research Department of Chemistry, Government Arts College, Coimbatore-18, Tamilnadu, India

**Keywords:** crystal structure, piperidine, diphenylpiperidine, 2-chloro-ethanone, hydrogen bonding, C—H⋯π interactions

## Abstract

In the title compound, C_20_H_22_ClNO, the piperidine ring has a twist-boat conformation. There is an intra­molecular C—H⋯π inter­action involving the two phenyl rings which are inclined to one another by 84.91 (7)°. In the crystal, mol­ecules are linked *via* C—H⋯O hydrogen bonds, forming helical chains along [010]. The chains are linked by C—H⋯π inter­actions, forming sheets parallel to (100).

## Related literature   

For the biological activity of piperidines and their derivatives, see: Aridoss *et al.* (2007 ▸); Jain *et al.* (2005 ▸); Mobio *et al.* (1989 ▸); Palani *et al.* (2002 ▸). For the crystal structure of a very similar compound, 2-chloro-1-(3,3-dimethyl-2,6-di­phenyl­piperidin-1-yl)ethanone, see: Prathebha *et al.* (2013 ▸).
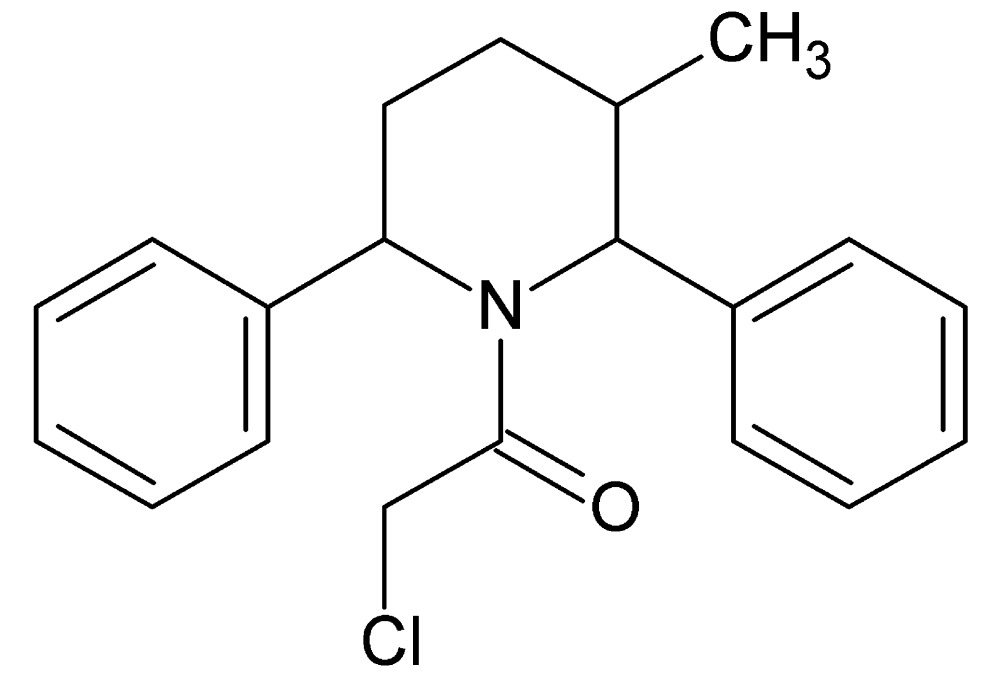


## Experimental   

### Crystal data  

C_20_H_22_ClNO
*M* *_r_* = 327.84
Monoclinic, 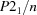

*a* = 8.7146 (3) Å
*b* = 12.3963 (4) Å
*c* = 16.6117 (6) Å
β = 101.523 (2)°
*V* = 1758.37 (10) Å^3^
*Z* = 4
Mo *K*α radiation
μ = 0.22 mm^−1^
*T* = 293 K
0.25 × 0.23 × 0.23 mm

### Data collection  

Bruker APEXII CCD diffractometer
Absorption correction: multi-scan (*SADABS*; Bruker, 2008 ▸) *T*_min_ = 0.946, *T*_max_ = 0.950
16473 measured reflections
4353 independent reflections
3075 reflections with *I* > 2σ(*I*)
*R*_int_ = 0.026

### Refinement  

*R*[*F* ^2^ > 2σ(*F* ^2^)] = 0.050
*wR*(*F* ^2^) = 0.236
*S* = 0.92
4353 reflections
208 parameters
H-atom parameters constrained
Δρ_max_ = 0.47 e Å^−3^
Δρ_min_ = −0.38 e Å^−3^

### 

Data collection: *APEX2* (Bruker, 2008 ▸); cell refinement: *SAINT* (Bruker, 2008 ▸); data reduction: *SAINT*; program(s) used to solve structure: *SHELXS97* (Sheldrick, 2008 ▸); program(s) used to refine structure: *SHELXL97* (Sheldrick, 2015 ▸); molecular graphics: *ORTEP-3 for Windows* (Farrugia, 2012 ▸); software used to prepare material for publication: *SHELXL97* and *PLATON* (Spek, 2009 ▸).

## Supplementary Material

Crystal structure: contains datablock(s) I, New_Global_Publ_Block. DOI: 10.1107/S205698901500122X/su5068sup1.cif

Structure factors: contains datablock(s) I. DOI: 10.1107/S205698901500122X/su5068Isup2.hkl

Supporting information file. DOI: 10.1107/S205698901500122X/su5068Isup3.cml

**Fig. 1.**
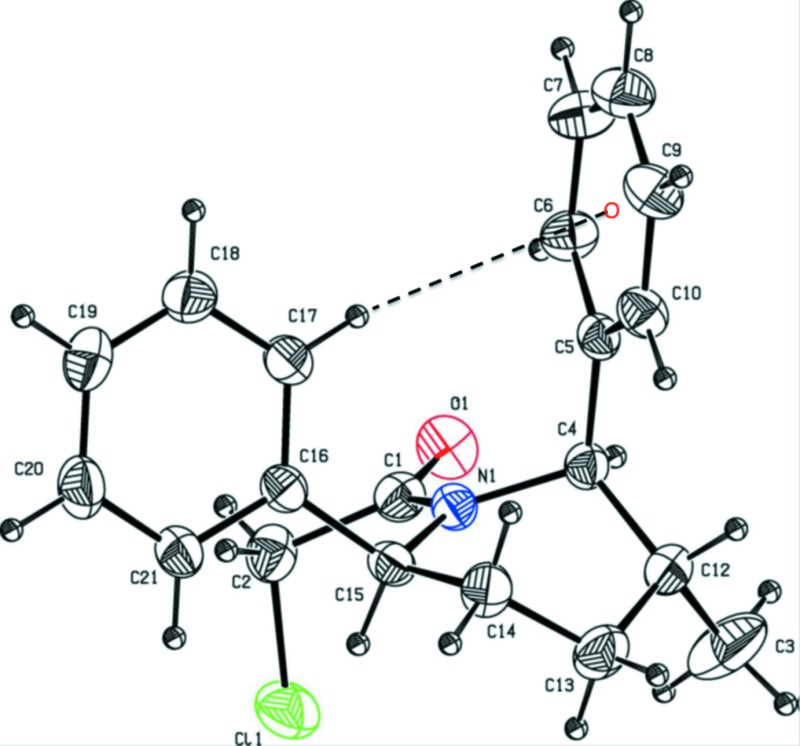
The molecular structure of the title compound, with atom labelling. Displacement ellipsoids are drawn at the 30% probability level. The intramolecular C—H···π interaction is shown as a dashed line (see Table 1 for details).

**Fig. 2.**
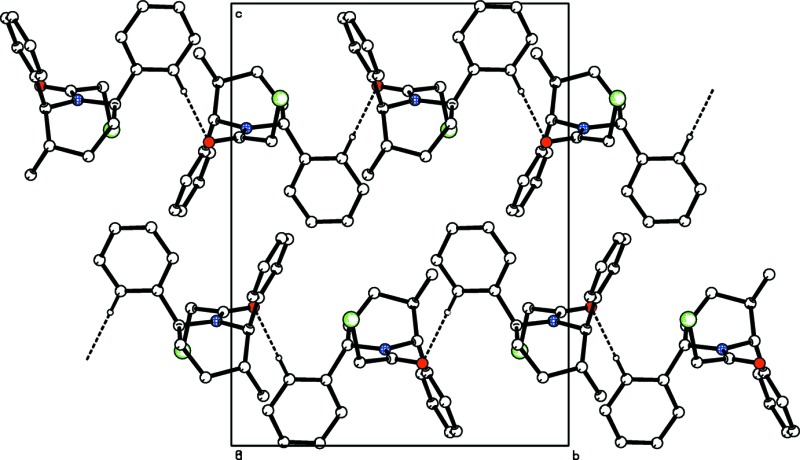
A view along the a axis of the crystal packing of the title compound. The dashed lines indicate the hydrogen bonds (see Table 1 for details).

. DOI: 10.1107/S205698901500122X/su5068fig1.tif
The mol­ecular structure of the title compound, with atom labelling. Displacement ellipsoids are drawn at the 30% probability level. The intra­molecular C—H⋯π inter­action is shown as a dashed line (see Table 1 for details).

a . DOI: 10.1107/S205698901500122X/su5068fig2.tif
A view along the *a* axis of the crystal packing of the title compound. The dashed lines indicate the hydrogen bonds (see Table 1 for details).

CCDC reference: 1044361

Additional supporting information:  crystallographic information; 3D view; checkCIF report

## Figures and Tables

**Table 1:** Hydrogen-bond geometry (, ) *Cg*1 is the centroid of the C5C10 ring.

*D*H*A*	*D*H	H*A*	*D* *A*	*D*H*A*
C17H17*Cg*1	0.93	2.98	3.879(2)	164
C21H21O1^i^	0.93	2.57	3.472(3)	165
C14H14b*Cg*1^ii^	0.98	2.84	3.751(2)	156

Symmetry codes: (i) 
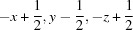
; (ii) 
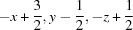
.

## supplementary crystallographic information

### S1. Structural commentary

Piperidine and their derivatives are significant heterocyclic compounds found in natural substances (Jain *et al.*, 2005). They have been observed to exhibit a wide range of biological activities, such as anti-fungal, anti-malarial, anti-bacterial and anti-viral activities (Aridoss *et al.*, 2007; Mobio *et al.*, 1989). They also show a highly favourable anti­viral activity against a range of primary HIV-1 isolates (Palani *et al.*, 2002).

The molecular structure of the title compound is illustrated in Fig. 1. The sum of the bond angles around atom N1 is 359.85 (1) °, indicating sp^2^ hybridization. The dihedral angle between the phenyl rings (C5—C10 and C16—C21) is 84.91 (7) °. They are linked by an intra­molecular C—H···π inter­action (Table 1). The piperidine ring adopts a twist-boat conformation.

In the crystal, molecules are linked via C—H···O hydrogen bonds forming helical chains along [010], see Table 1 and Fig. 2. The chains are linked by C—H···π inter­actions forming sheets parallel to (100), see Table 1.

### S2. Synthesis and crystallization

A mixture of t-3-methyl-r-2,c-6-di­phenyl­piperidine (5 mmol), chloro­acetyl chloride (20 mmol) and tri­ethyl­amine (20 mmol) in anhydrous benzene (20 ml) was stirred at rt. The precipitated ammonium salt was filtered and the resulting solution was washed with water and bicarbonate solution (4 × 10 ml). Finally, the benzene solution was dried over anhydrous sodium sulfate and concentrated. The pasty mass was purified by crystallization from pet-ether (333-353 K) and ethyl acetate in the ratio of 95: 5, and yielded colourless block-like crystals.

### S3. Refinement

Crystal data, data collection and structure refinement details are summarized in Table 1. H atoms were positioned geometrically and treated as riding on their parent atoms: C—H = 0.93 - 0.98 Å, with *U*_iso_(H)= 1.5 *U*_eq_(C) for methyl H atoms and = 1.2U_eq_(C) for other H atoms.

### Crystal data

**Table:**

C_20_H_22_ClNO	*F*(000) = 696
*M**_r_* = 327.84	*D*_x_ = 1.238 Mg m^−^^3^
Monoclinic, *P*2_1_/*n*	Mo *K*α radiation, λ = 0.71073 Å
Hall symbol: -P 2yn	Cell parameters from 4353 reflections
*a* = 8.7146 (3) Å	θ = 2.1–28.2°
*b* = 12.3963 (4) Å	µ = 0.22 mm^−^^1^
*c* = 16.6117 (6) Å	*T* = 293 K
β = 101.523 (2)°	Block, colourless
*V* = 1758.37 (10) Å^3^	0.25 × 0.23 × 0.23 mm
*Z* = 4	

### Data collection

**Table:**

Bruker APEXII CCD diffractometer	4353 independent reflections
Radiation source: fine-focus sealed tube	3075 reflections with *I* > 2σ(*I*)
Graphite monochromator	*R*_int_ = 0.026
ω and φ scan	θ_max_ = 28.2°, θ_min_ = 2.1°
Absorption correction: multi-scan (*SADABS*; Bruker, 2008)	*h* = −11→11
*T*_min_ = 0.946, *T*_max_ = 0.950	*k* = −16→14
16473 measured reflections	*l* = −22→16

### Refinement

**Table:**

Refinement on *F*^2^	Primary atom site location: structure-invariant direct methods
Least-squares matrix: full	Secondary atom site location: difference Fourier map
*R*[*F*^2^ > 2σ(*F*^2^)] = 0.050	Hydrogen site location: inferred from neighbouring sites
*wR*(*F*^2^) = 0.236	H-atom parameters constrained
*S* = 0.92	*w* = 1/[σ^2^(*F*_o_^2^) + (0.2*P*)^2^] where *P* = (*F*_o_^2^ + 2*F*_c_^2^)/3
4353 reflections	(Δ/σ)_max_ < 0.001
208 parameters	Δρ_max_ = 0.47 e Å^−^^3^
0 restraints	Δρ_min_ = −0.38 e Å^−^^3^

### Special details

**Table:**

Geometry. All e.s.d.'s (except the e.s.d. in the dihedral angle between two l.s. planes) are estimated using the full covariance matrix. The cell e.s.d.'s are taken into account individually in the estimation of e.s.d.'s in distances, angles and torsion angles; correlations between e.s.d.'s in cell parameters are only used when they are defined by crystal symmetry. An approximate (isotropic) treatment of cell e.s.d.'s is used for estimating e.s.d.'s involving l.s. planes.
Refinement. Refinement of *F*^2^ against ALL reflections. The weighted *R*-factor *wR* and goodness of fit *S* are based on *F*^2^, conventional *R*-factors *R* are based on *F*, with *F* set to zero for negative *F*^2^. The threshold expression of *F*^2^ > σ(*F*^2^) is used only for calculating *R*-factors(gt) *etc*. and is not relevant to the choice of reflections for refinement. *R*-factors based on *F*^2^ are statistically about twice as large as those based on *F*, and *R*- factors based on ALL data will be even larger.

### Fractional atomic coordinates and isotropic or equivalent isotropic displacement parameters (Å^2^)

**Table:**

	*x*	*y*	*z*	*U*_iso_*/*U*_eq_	
C17	0.6269 (2)	0.82630 (15)	0.42412 (12)	0.0533 (5)	
H17	0.6507	0.8995	0.4264	0.064*	
C1	0.3083 (2)	0.97610 (15)	0.30256 (11)	0.0498 (4)	
C2	0.2026 (2)	0.88094 (17)	0.31112 (13)	0.0579 (5)	
H2A	0.1400	0.8972	0.3517	0.069*	
H2B	0.2657	0.8177	0.3293	0.069*	
C3	0.4639 (5)	1.0949 (3)	0.11267 (17)	0.1078 (11)	
H3A	0.4508	1.1680	0.1290	0.162*	
H3B	0.3657	1.0575	0.1062	0.162*	
H3C	0.4991	1.0943	0.0615	0.162*	
C4	0.53945 (19)	1.05108 (12)	0.26296 (10)	0.0426 (4)	
H4	0.4660	1.1118	0.2571	0.051*	
C5	0.67052 (19)	1.08102 (12)	0.33476 (10)	0.0415 (4)	
C6	0.6292 (2)	1.13399 (16)	0.40066 (12)	0.0548 (5)	
H6	0.5240	1.1470	0.4003	0.066*	
C7	0.7409 (3)	1.16786 (19)	0.46692 (14)	0.0678 (6)	
H7	0.7102	1.2039	0.5102	0.081*	
C8	0.8963 (3)	1.14864 (17)	0.46926 (15)	0.0674 (6)	
H8	0.9713	1.1707	0.5142	0.081*	
C9	0.9404 (2)	1.09646 (16)	0.40468 (14)	0.0615 (5)	
H9	1.0458	1.0829	0.4061	0.074*	
C10	0.8292 (2)	1.06365 (14)	0.33729 (12)	0.0507 (4)	
H10	0.8610	1.0297	0.2934	0.061*	
C12	0.5843 (2)	1.03897 (16)	0.17806 (11)	0.0548 (5)	
H12	0.6843	1.0764	0.1807	0.066*	
C13	0.6075 (3)	0.92372 (16)	0.15453 (13)	0.0677 (6)	
H13A	0.5134	0.8986	0.1178	0.081*	
H13B	0.6929	0.9205	0.1250	0.081*	
C14	0.6440 (3)	0.84909 (14)	0.22878 (13)	0.0555 (5)	
H14A	0.7360	0.8750	0.2666	0.067*	
H14B	0.6661	0.7772	0.2111	0.067*	
C15	0.5053 (2)	0.84491 (12)	0.27212 (11)	0.0446 (4)	
H15	0.4208	0.8059	0.2360	0.053*	
C16	0.54725 (18)	0.78122 (13)	0.35133 (10)	0.0439 (4)	
C18	0.6713 (2)	0.76237 (18)	0.49382 (13)	0.0601 (5)	
H18	0.7253	0.7932	0.5423	0.072*	
C19	0.6364 (3)	0.65523 (18)	0.49162 (15)	0.0633 (5)	
H19	0.6673	0.6130	0.5383	0.076*	
C20	0.5549 (3)	0.60920 (16)	0.41981 (15)	0.0660 (6)	
H20	0.5300	0.5362	0.4184	0.079*	
C21	0.5101 (2)	0.67207 (14)	0.34978 (13)	0.0533 (5)	
H21	0.4551	0.6410	0.3016	0.064*	
N1	0.44797 (15)	0.95570 (11)	0.28196 (8)	0.0429 (4)	
O1	0.25929 (16)	1.06617 (12)	0.31359 (10)	0.0666 (4)	
Cl1	0.07878 (7)	0.85512 (5)	0.21470 (4)	0.0825 (3)	

### Atomic displacement parameters (Å^2^)

**Table:**

	*U*^11^	*U*^22^	*U*^33^	*U*^12^	*U*^13^	*U*^23^
C17	0.0562 (11)	0.0472 (10)	0.0536 (11)	−0.0082 (8)	0.0041 (8)	0.0001 (8)
C1	0.0431 (9)	0.0574 (10)	0.0479 (9)	0.0011 (7)	0.0069 (7)	0.0009 (8)
C2	0.0448 (9)	0.0684 (12)	0.0604 (12)	−0.0021 (8)	0.0106 (8)	0.0106 (9)
C3	0.156 (3)	0.112 (2)	0.0510 (14)	0.046 (2)	0.0100 (16)	0.0171 (14)
C4	0.0463 (8)	0.0389 (8)	0.0431 (9)	0.0000 (6)	0.0101 (7)	0.0021 (6)
C5	0.0471 (8)	0.0327 (7)	0.0455 (9)	−0.0017 (6)	0.0110 (7)	0.0004 (6)
C6	0.0509 (10)	0.0632 (11)	0.0518 (11)	0.0061 (8)	0.0138 (8)	−0.0084 (8)
C7	0.0716 (14)	0.0793 (14)	0.0506 (11)	0.0073 (11)	0.0079 (10)	−0.0165 (10)
C8	0.0613 (12)	0.0715 (13)	0.0623 (13)	−0.0037 (10)	−0.0046 (10)	−0.0086 (10)
C9	0.0477 (10)	0.0570 (11)	0.0772 (14)	−0.0019 (8)	0.0062 (9)	−0.0096 (10)
C10	0.0494 (9)	0.0437 (9)	0.0601 (11)	−0.0015 (7)	0.0139 (8)	−0.0070 (8)
C12	0.0668 (12)	0.0546 (11)	0.0447 (10)	−0.0038 (8)	0.0151 (8)	0.0025 (8)
C13	0.0993 (16)	0.0601 (12)	0.0502 (11)	0.0033 (11)	0.0305 (11)	−0.0054 (9)
C14	0.0698 (12)	0.0449 (9)	0.0578 (11)	0.0054 (8)	0.0269 (9)	−0.0036 (8)
C15	0.0472 (9)	0.0385 (8)	0.0465 (9)	−0.0035 (6)	0.0057 (7)	−0.0045 (6)
C16	0.0395 (8)	0.0427 (8)	0.0496 (9)	−0.0013 (6)	0.0089 (7)	−0.0017 (7)
C18	0.0521 (10)	0.0715 (13)	0.0528 (11)	−0.0055 (9)	0.0014 (8)	0.0043 (9)
C19	0.0608 (12)	0.0658 (12)	0.0648 (13)	0.0057 (9)	0.0159 (10)	0.0212 (10)
C20	0.0820 (15)	0.0449 (10)	0.0749 (14)	−0.0032 (9)	0.0245 (12)	0.0103 (9)
C21	0.0600 (11)	0.0424 (9)	0.0580 (11)	−0.0060 (7)	0.0134 (9)	−0.0014 (8)
N1	0.0422 (7)	0.0418 (7)	0.0445 (8)	−0.0020 (5)	0.0081 (6)	0.0003 (6)
O1	0.0523 (8)	0.0640 (9)	0.0863 (11)	0.0095 (6)	0.0205 (7)	−0.0041 (7)
Cl1	0.0729 (4)	0.0820 (5)	0.0812 (5)	−0.0188 (3)	−0.0121 (3)	−0.0008 (3)

### Geometric parameters (Å, º)

**Table:**

C17—C16	1.386 (2)	C8—H8	0.9300
C17—C18	1.393 (3)	C9—C10	1.387 (3)
C17—H17	0.9300	C9—H9	0.9300
C1—O1	1.222 (2)	C10—H10	0.9300
C1—N1	1.352 (2)	C12—C13	1.505 (3)
C1—C2	1.521 (3)	C12—H12	0.9800
C2—Cl1	1.773 (2)	C13—C14	1.524 (3)
C2—H2A	0.9700	C13—H13A	0.9700
C2—H2B	0.9700	C13—H13B	0.9700
C3—C12	1.518 (3)	C14—C15	1.526 (3)
C3—H3A	0.9600	C14—H14A	0.9700
C3—H3B	0.9600	C14—H14B	0.9700
C3—H3C	0.9600	C15—N1	1.482 (2)
C4—N1	1.495 (2)	C15—C16	1.515 (2)
C4—C5	1.523 (2)	C15—H15	0.9800
C4—C12	1.544 (2)	C16—C21	1.390 (2)
C4—H4	0.9800	C18—C19	1.361 (3)
C5—C6	1.384 (2)	C18—H18	0.9300
C5—C10	1.392 (2)	C19—C20	1.384 (3)
C6—C7	1.381 (3)	C19—H19	0.9300
C6—H6	0.9300	C20—C21	1.390 (3)
C7—C8	1.368 (3)	C20—H20	0.9300
C7—H7	0.9300	C21—H21	0.9300
C8—C9	1.372 (3)		
			
C16—C17—C18	120.23 (17)	C13—C12—C3	110.9 (2)
C16—C17—H17	119.9	C13—C12—C4	113.64 (15)
C18—C17—H17	119.9	C3—C12—C4	110.11 (19)
O1—C1—N1	124.60 (17)	C13—C12—H12	107.3
O1—C1—C2	117.28 (17)	C3—C12—H12	107.3
N1—C1—C2	118.11 (16)	C4—C12—H12	107.3
C1—C2—Cl1	109.05 (13)	C14—C13—C12	112.53 (17)
C1—C2—H2A	109.9	C14—C13—H13A	109.1
Cl1—C2—H2A	109.9	C12—C13—H13A	109.1
C1—C2—H2B	109.9	C14—C13—H13B	109.1
Cl1—C2—H2B	109.9	C12—C13—H13B	109.1
H2A—C2—H2B	108.3	H13A—C13—H13B	107.8
C12—C3—H3A	109.5	C13—C14—C15	110.24 (17)
C12—C3—H3B	109.5	C13—C14—H14A	109.6
H3A—C3—H3B	109.5	C15—C14—H14A	109.6
C12—C3—H3C	109.5	C13—C14—H14B	109.6
H3A—C3—H3C	109.5	C15—C14—H14B	109.6
H3B—C3—H3C	109.5	H14A—C14—H14B	108.1
N1—C4—C5	112.06 (13)	N1—C15—C16	114.54 (14)
N1—C4—C12	111.04 (13)	N1—C15—C14	109.68 (13)
C5—C4—C12	116.84 (14)	C16—C15—C14	110.55 (15)
N1—C4—H4	105.3	N1—C15—H15	107.2
C5—C4—H4	105.3	C16—C15—H15	107.2
C12—C4—H4	105.3	C14—C15—H15	107.2
C6—C5—C10	117.60 (16)	C17—C16—C21	118.86 (17)
C6—C5—C4	117.51 (15)	C17—C16—C15	122.65 (15)
C10—C5—C4	124.82 (15)	C21—C16—C15	118.43 (15)
C7—C6—C5	121.42 (18)	C19—C18—C17	120.6 (2)
C7—C6—H6	119.3	C19—C18—H18	119.7
C5—C6—H6	119.3	C17—C18—H18	119.7
C8—C7—C6	120.4 (2)	C18—C19—C20	119.92 (19)
C8—C7—H7	119.8	C18—C19—H19	120.0
C6—C7—H7	119.8	C20—C19—H19	120.0
C7—C8—C9	119.4 (2)	C19—C20—C21	120.01 (18)
C7—C8—H8	120.3	C19—C20—H20	120.0
C9—C8—H8	120.3	C21—C20—H20	120.0
C8—C9—C10	120.65 (19)	C20—C21—C16	120.33 (19)
C8—C9—H9	119.7	C20—C21—H21	119.8
C10—C9—H9	119.7	C16—C21—H21	119.8
C5—C10—C9	120.56 (18)	C1—N1—C15	122.79 (14)
C5—C10—H10	119.7	C1—N1—C4	116.78 (14)
C9—C10—H10	119.7	C15—N1—C4	120.28 (13)
			
O1—C1—C2—Cl1	88.83 (19)	C18—C17—C16—C15	−175.86 (17)
N1—C1—C2—Cl1	−90.01 (18)	N1—C15—C16—C17	−41.6 (2)
N1—C4—C5—C6	−75.98 (19)	C14—C15—C16—C17	82.9 (2)
C12—C4—C5—C6	154.28 (16)	N1—C15—C16—C21	141.29 (16)
N1—C4—C5—C10	107.24 (18)	C14—C15—C16—C21	−94.20 (19)
C12—C4—C5—C10	−22.5 (2)	C16—C17—C18—C19	−0.4 (3)
C10—C5—C6—C7	−0.4 (3)	C17—C18—C19—C20	−0.5 (3)
C4—C5—C6—C7	−177.46 (19)	C18—C19—C20—C21	0.7 (3)
C5—C6—C7—C8	−0.7 (3)	C19—C20—C21—C16	0.2 (3)
C6—C7—C8—C9	0.8 (4)	C17—C16—C21—C20	−1.1 (3)
C7—C8—C9—C10	0.3 (3)	C15—C16—C21—C20	176.11 (18)
C6—C5—C10—C9	1.5 (3)	O1—C1—N1—C15	177.72 (17)
C4—C5—C10—C9	178.23 (16)	C2—C1—N1—C15	−3.5 (3)
C8—C9—C10—C5	−1.4 (3)	O1—C1—N1—C4	−6.8 (3)
N1—C4—C12—C13	−30.7 (2)	C2—C1—N1—C4	171.99 (14)
C5—C4—C12—C13	99.5 (2)	C16—C15—N1—C1	−69.0 (2)
N1—C4—C12—C3	94.4 (2)	C14—C15—N1—C1	166.04 (16)
C5—C4—C12—C3	−135.4 (2)	C16—C15—N1—C4	115.63 (16)
C3—C12—C13—C14	−146.8 (2)	C14—C15—N1—C4	−9.3 (2)
C4—C12—C13—C14	−22.1 (3)	C5—C4—N1—C1	101.09 (17)
C12—C13—C14—C15	63.8 (2)	C12—C4—N1—C1	−126.23 (17)
C13—C14—C15—N1	−46.3 (2)	C5—C4—N1—C15	−83.27 (17)
C13—C14—C15—C16	−173.53 (16)	C12—C4—N1—C15	49.41 (19)
C18—C17—C16—C21	1.3 (3)		

### Hydrogen-bond geometry (Å, º)

Cg1 is the centroid of the C5–C10 ring.

**Table:**

*D*—H···*A*	*D*—H	H···*A*	*D*···*A*	*D*—H···*A*
C17—H17···*Cg*1	0.93	2.98	3.879 (2)	164
C21—H21···O1^i^	0.93	2.57	3.472 (3)	165
C14—H14b···*Cg*1^ii^	0.98	2.84	3.751 (2)	156

Symmetry codes: (i) −*x*+1/2, *y*−1/2, −*z*+1/2; (ii) −*x*+3/2, *y*−1/2, −*z*+1/2.
