# Supplementary figures and images for: Crystal structure of 2-chloro-1-(3-methyl-2,6-di­phenyl­piperidin-1-yl)ethanone
Source: Acta Crystallogr E Crystallogr Commun. 2015 Jan 28;71(Pt 2):o135–6. doi: 10.1107/S205698901500122X (PMC4384538; doi:10.1107/S205698901500122X)

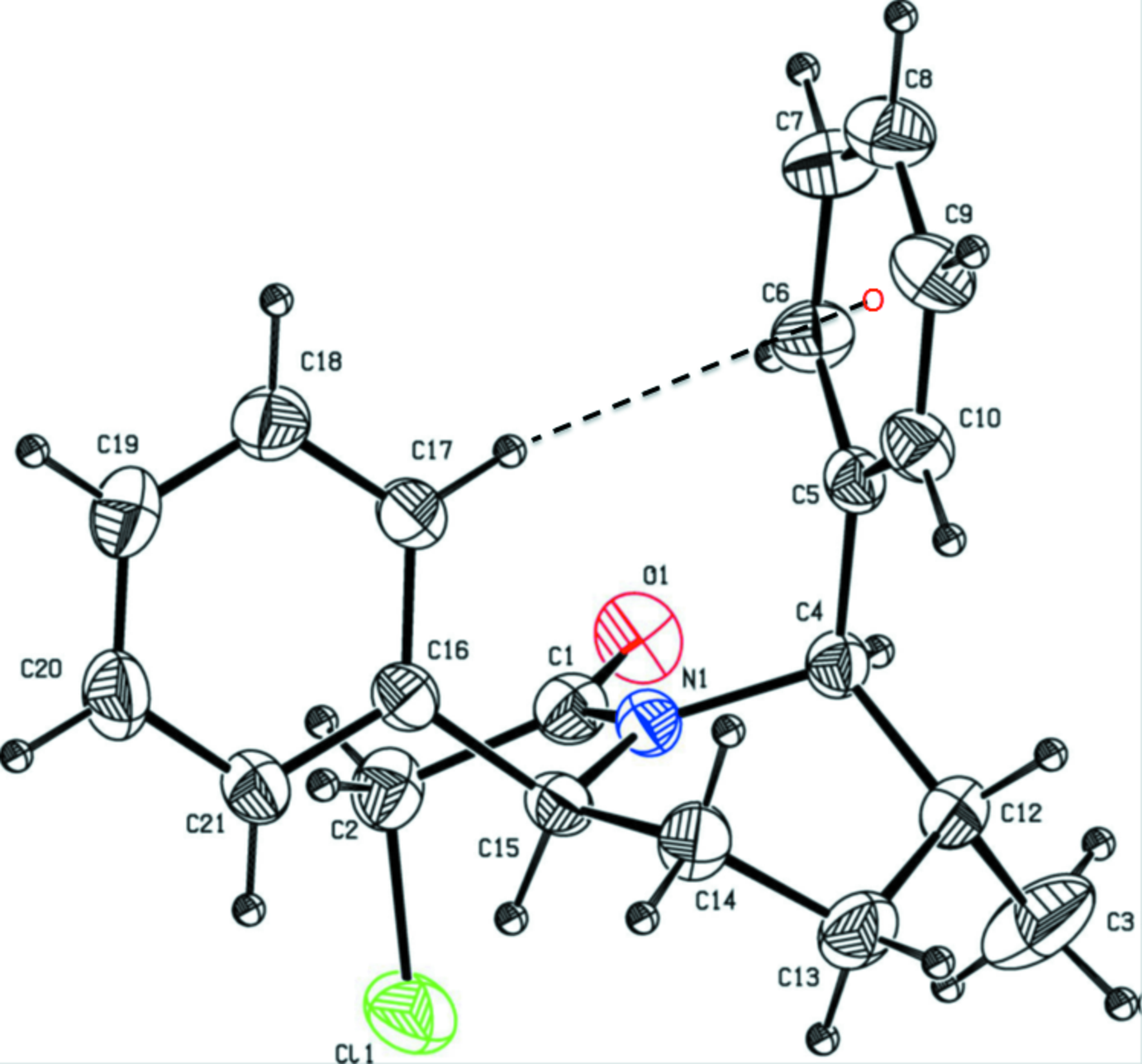

Supplement: Supplementary file 4 [file e-71-0o135-fig1.tif]

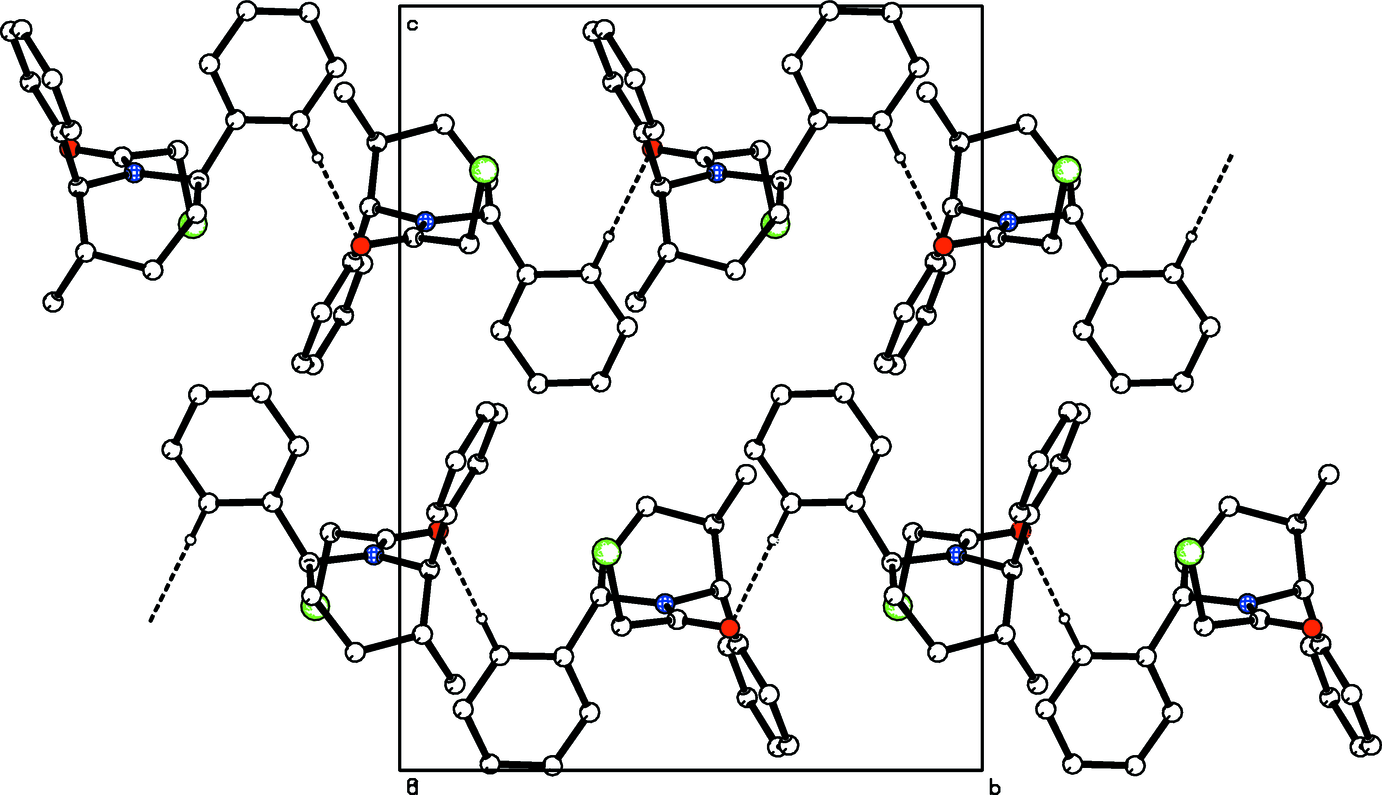

Supplement: Supplementary file 5 [file e-71-0o135-fig2.tif]
